# Supplementary material for: PCR enables rapid detection of dermatophytes in practice
Source: Microbiol Spectr. 2024 Sep 17;12(11):e01049-24. doi: 10.1128/spectrum.01049-24 (PMC11537052; doi:10.1128/spectrum.01049-24)
Supplement: Table S1 — DermaGenius PCR validation data. [file spectrum.01049-24-s0001.docx]

Supplementary Table 1. Results of DermaGenius 2.0 (DG) verification: a comparison of culture and DG-PCR methods.

| **Sample** | **Species by culture** | **Species by DG-PCR** |
| --- | --- | --- |
| ATCC 10231 | C. albicans | C. albicans |
| ATCC 28942 | T. tonsurans | T. tonsurans |
| ATCC 9533 | T. interdigitale | T. interdigitale |
| ATCC 28188 | T. rubrum | T. rubrum |
| QC1 | E. floccosum | E. floccosum |
| QC2 | M. canis | M. canis |
| QC3 | M. audouinii | M. audouinii |
| QC4 | T. mentagrophytes | T. mentagrophytes |
| QC5 | T. soudanense | T. soudanense |
| QC6 | T. benhamiae | T. benhamiae |
| QC7 | T. verrucosum | T. verrucosum |
| Patient sample 1 | T. mentagrophytes | T. mentagrophytes |
| Patient sample 2 | T. violaceum | T. violaceum |
| Patient sample 3 | T. rubrum | T. rubrum |
| Patient sample 4 | T. verrucosum | T. verrucosum |
| Patient sample 5 | Trichophyton sp. (T. verrucosum/T. erinacei, atypical strain) | T. verrucosum |
| QC = UK NEQAS/Mycology scheme | |  |
